# Clinlabomics: leveraging clinical laboratory data by data mining strategies

**DOI:** 10.1186/s12859-022-04926-1

**Published:** 2022-09-24

**Authors:** Xiaoxia Wen, Ping Leng, Jiasi Wang, Guishu Yang, Ruiling Zu, Xiaojiong Jia, Kaijiong Zhang, Birga Anteneh Mengesha, Jian Huang, Dongsheng Wang, Huaichao Luo

**Affiliations:** 1 Department of Clinical Laboratory, Sichuan Cancer Hospital & Institute, Sichuan Cancer Center, School of Medicine, University of Electronic Science and Technology of China, Chengdu, Sichuan China; 2 College of Medical Technology, Chengdu University of Traditional Chinese Medicine, Chengdu, Sichuan China; 3 Department of Clinical Laboratory, Dazhou Central Hospital, Dazhou, Sichuan China; 4 School of Life Science and Technology, University of Electronic Science and Technology of China, Chengdu, Sichuan China; 5 Department of Laboratory Medicine, Chongqing Health Center for Women and Children, Yubei District, Chongqing, China

**Keywords:** Clinlabomics, Data mining, Artificial intelligence, Clinical laboratory, Machine learning, Deep learning, Data science, Medical laboratory science

## Abstract

The recent global focus on big data in medicine has been associated with the rise of artificial intelligence (AI) in diagnosis and decision-making following recent advances in computer technology. Up to now, AI has been applied to various aspects of medicine, including disease diagnosis, surveillance, treatment, predicting future risk, targeted interventions and understanding of the disease. There have been plenty of successful examples in medicine of using big data, such as radiology and pathology, ophthalmology cardiology and surgery. Combining medicine and AI has become a powerful tool to change health care, and even to change the nature of disease screening in clinical diagnosis. As all we know, clinical laboratories produce large amounts of testing data every day and the clinical laboratory data combined with AI may establish a new diagnosis and treatment has attracted wide attention. At present, a new concept of radiomics has been created for imaging data combined with AI, but a new definition of clinical laboratory data combined with AI has lacked so that many studies in this field cannot be accurately classified. Therefore, we propose a new concept of clinical laboratory omics (Clinlabomics) by combining clinical laboratory medicine and AI. Clinlabomics can use high-throughput methods to extract large amounts of feature data from blood, body fluids, secretions, excreta, and cast clinical laboratory test data. Then using the data statistics, machine learning, and other methods to read more undiscovered information. In this review, we have summarized the application of clinical laboratory data combined with AI in medical fields. Undeniable, the application of Clinlabomics is a method that can assist many fields of medicine but still requires further validation in a multi-center environment and laboratory.

## Introduction

**Fig. 1:**
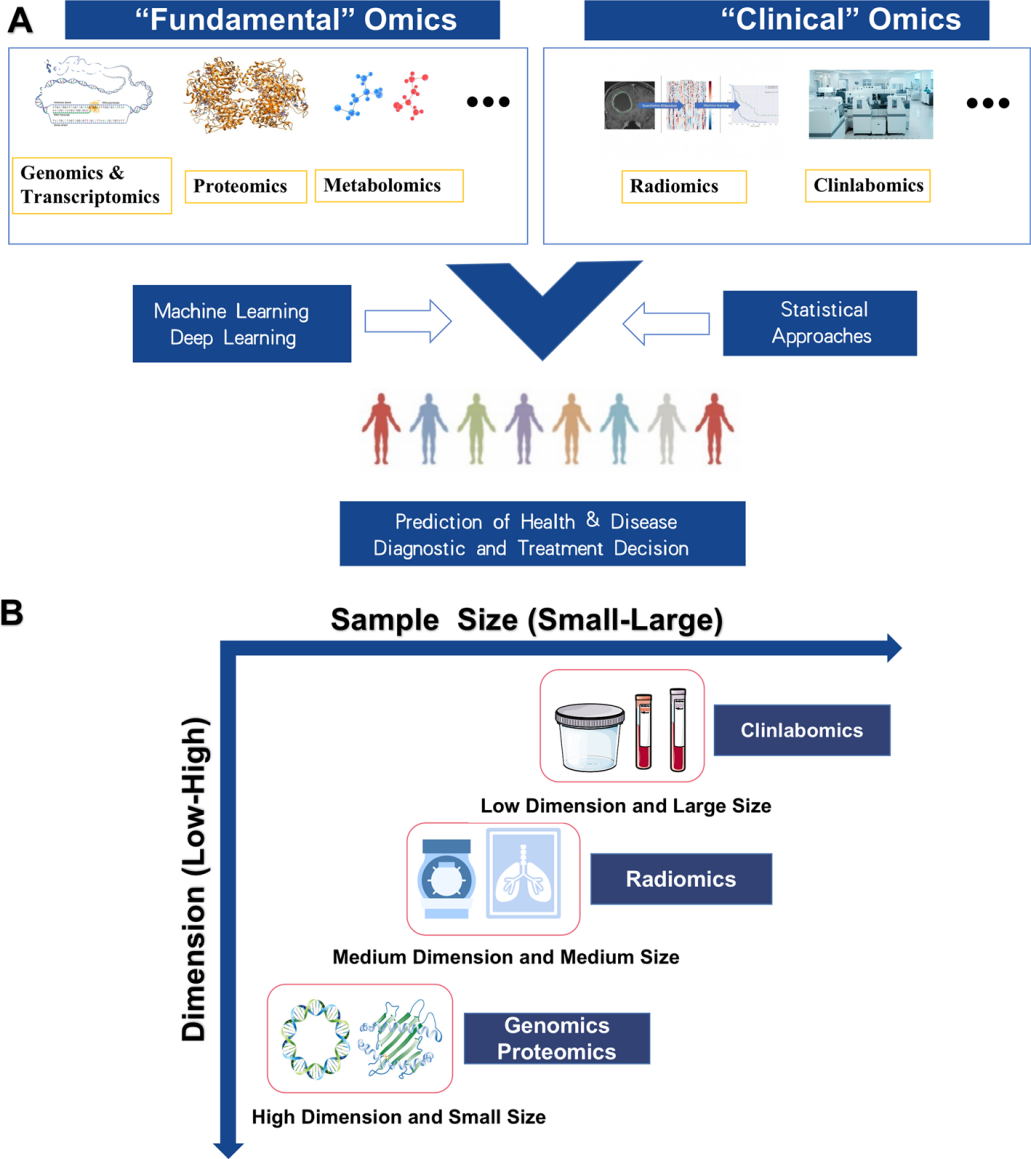
**A** The technology of “omics” e.g. genomics, proteomics, transcriptomics, metabolomics radiomics etc. can be used for more accurate predicting and understanding disease risks and formulating treatments for more specific and homogeneous populations by machine learning and statistical approaches. **B** Differences in the data structure between the different omics

The technology of “omics” (genomics, proteomics, transcriptomics, metabolomics, etc.) is an emerging practice. We can more accurately predict and understand disease risks and formulate treatments for more specific and homogeneous populations by using big data, technologies, and methods [1, 2] (Fig. 1A). Since the advent of high-throughput and ultra-high-throughput sequencing technologies, clinicians and molecular biologists share the concern of discovering individual differences in disease based on the individual genome and transcriptome, which enables better clinical management [3–5]. Due to the discovery of specific proteins associated with human disease, the field of protein chemistry and subsequent proteomics devote to the search for new or better disease markers and therapeutic targets [6, 7]. In addition to the development of fundamental omics, clinical omics are also improving. For example, the application of emerging radiomics supported personalized clinical decisions and individualized treatment choices. A high-throughput method was used to extract and analyze a large number of image features from radiographic images to develop diagnostic, predictive, or prognostic imaging models [8, 9].

**Fig. 2:**
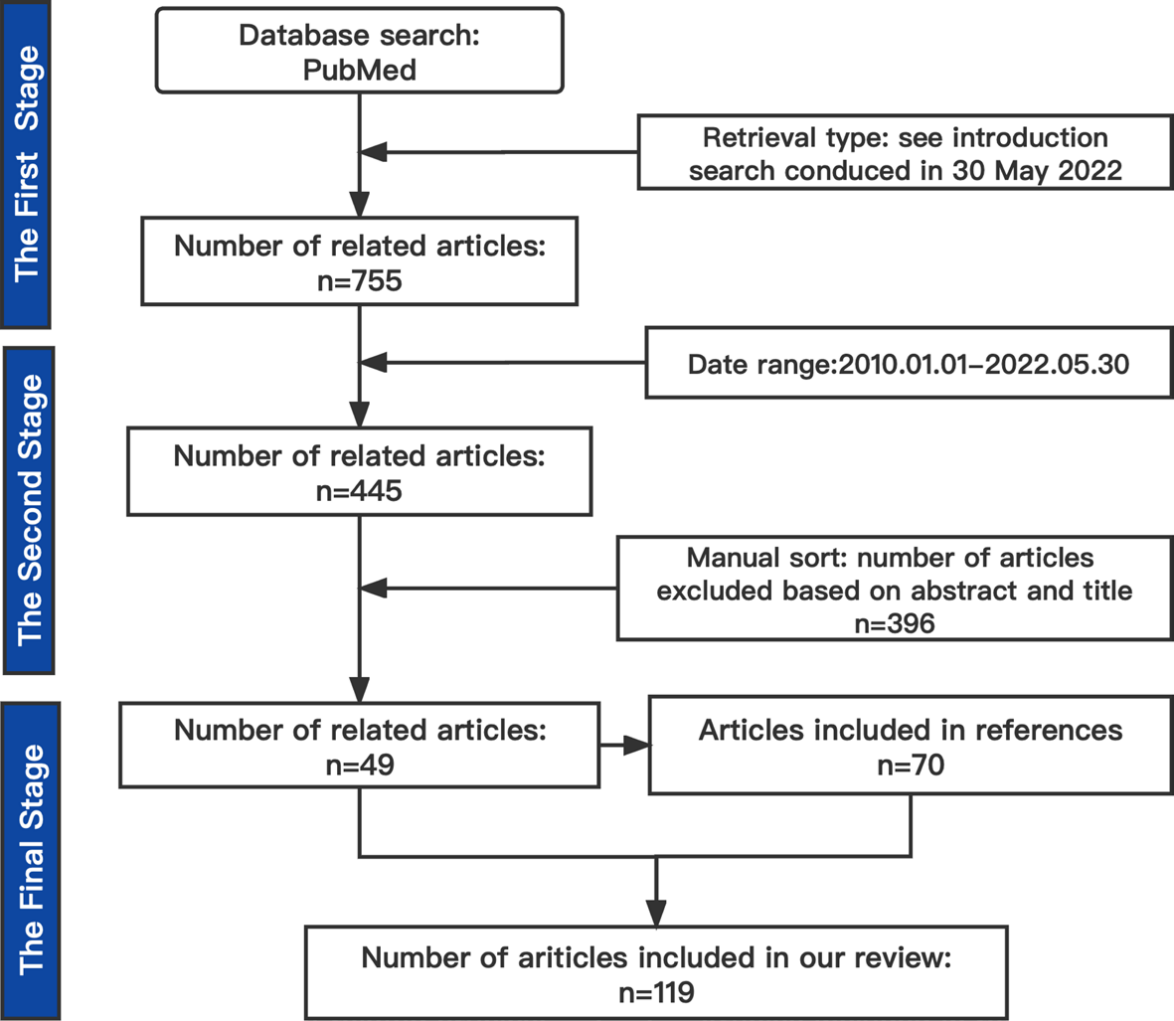
The workflow for searching and filtering articles

The clinical laboratory is the department that provides valuable test results and auxiliary clinical diagnosis by using visual observation, physical, biochemical, or molecular biological methods to examine specimens of patients, such as blood, urine, effusion, and tumor tissue [10]. Although current clinical laboratory testing indexes are less than the variables of transcriptomics, genomics, and even radiomics. The data dimension of Clinlabomics is lower than other omics. However, the clinical laboratory produces a large amount of data every day and many diseases currently need the assistance of the clinical laboratory test results. Besides, the clinical laboratory data also is quantitative. The clinical data collected by the clinical laboratory are larger and more intuitive than the imaging data (Fig. 1B). Therefore, we speculate that it is possible to develop clinical laboratory medicine omics models for promoting the development of all areas of medicine by integrating the test data information. By 2021, searches using "AI" and "Medicine" in PubMed would produce nearly 6,000 articles compared to 4,000 articles in 2020. We used search pattern: ("Artificial Intelligence"[Title/Abstract] OR "Artificial Intelligence"[MeSH Terms] OR "Machine Learning"[Title/Abstract] OR "Machine Learning"[MeSH Terms] OR "Data Mining"[Title/Abstract] OR "Data Mining"[MeSH Terms] OR "Deep Learning"[MeSH Terms] OR "Big Data"[MeSH Terms] OR "Big Data"[Title/Abstract] OR "Deep Learning"[Title/Abstract] OR "Data Science"[MeSH Terms] OR "Data Science"[Title/Abstract]) AND ("clinical laboratory"[Title/Abstract] OR "laboratories, clinical"[MeSH Terms] OR "clinical laboratories"[Title/Abstract] OR "laboratory medicine"[Title/Abstract] OR "Medical Laboratory Science"[MeSH Terms] OR "Clinical Laboratory Information Systems"[MeSH Terms] OR "laboratory science medical"[Title/Abstract] OR "clinical biochemistry"[Title/Abstract] OR "blood routine"[Title/Abstract] OR "urine routine"[Title/Abstract] OR "coagulation test"[Title/Abstract] OR "pretransfusion tests"[Title/Abstract] OR "clinical immunoassay"[Title/Abstract] OR "Blood Coagulation Tests"[MeSH Terms] OR "clinical microbiology"[Title/Abstract]) to retrieval relevant articles in PubMed from 2010 to 2022 and approximately got 445 papers. Through manual title and abstract review, we finally identify related articles of the clinical laboratory in combination with AI and some articles in references for review and the workflow see Fig. 2. We excluded some laboratory work that did not belong to the hospital clinical laboratory department combined with AI work, including pathology, iconography and other laboratories combined with AI work research.

## Progress in clinical laboratory medicine

**Fig. 3:**
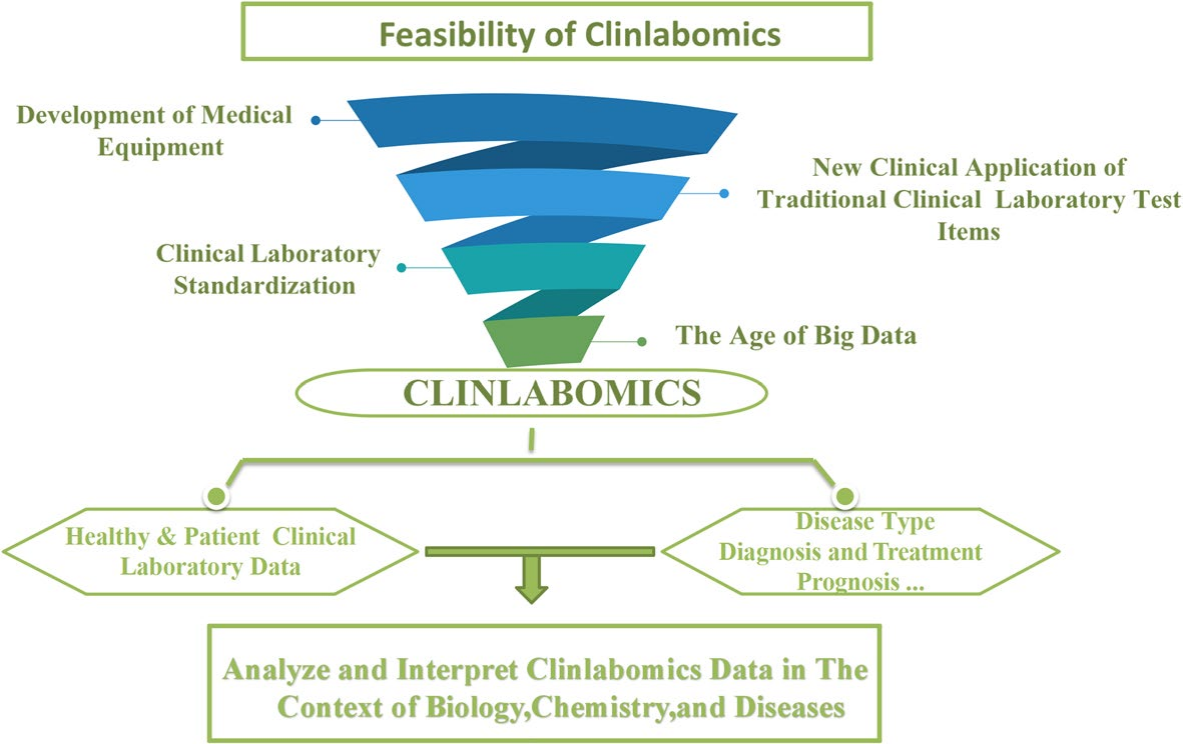
The development of the time has created conditions for the establishment of Clinlabomics. Mainly include the advantage of the development of clinical laboratory and the coming of the era of big data

The development of laboratory technology has created conditions for the establishment of Clinlabomics (Fig. 3). In the past decade, clinical laboratory medicine has progressed in four distinct areas.

### Development of medical equipment

Before the 1980s, clinical laboratory equipment was relatively straightforward. And this situation led to the type of clinical laboratory test indexes being limited [11]. Whereas, with the continuous development and the progress of society and science, clinical laboratory medicine has reached an unprecedented prosperous stage from the era of manual medical tests to semi-automatic and full-automatic analysis. Now, clinical blood, biochemistry, and microbial testing in the clinical laboratory have been automated [12–14]. Automated equipment rapidly and efficiently increased the throughput of a laboratory and has enabled us to monitor and manage the raw data produced more effectively than before [11, 15]. In brief, the renewal of laboratory equipment increases the efficiency of clinical tests so that we can get more data from clinical laboratory testing.

### Clinical laboratory standardization

For clinical laboratory test data, the quality of the data is as crucial as the quantity of data [16, 17]. In the past, most medical institutions carried out clinical laboratory tests by medical institutions themselves and there was no global quality assurance guideline. Until the international quality assurance of the ISO15189 standard was accepted. The ISO15189 is still the internationally accepted standard that has high credibility in quality management systems for all fields of laboratory medicine [18, 19]. The standardization of clinical laboratory test methods and the unified quality control standards make the clinical laboratory test results of different clinical laboratories more comparable [20]. A correct diagnosis and treatment decision is based on accurate clinical laboratory test results.

### New clinical significance of traditional test items

The sensitivity and specificity of different clinical laboratory test indexes for diagnosing a particular disease are different, so each item has its default clinical significance. In addition to the further study of various diseases and a deeper understanding of the physiological and pathological changes caused by diseases, some conventional clinical laboratory items have been found to possess more undiscovered clinical significance. For example, platelets were widely known to play a key role in hemostasis and thrombosis disorders [21, 22]. But now, it is known that platelets also contribute to immune and inflammatory activities in health and disease, including cancer progression [23–25]. For many years, prealbumin has been used as a measure of body nutritional status [26]. Now, the prognostic role of prealbumin in some tumor patients is recognized. A Study proved that for early relapse lung cancer patients, perioperative serum prealbumin levels were significantly lower than those in non-recurrence lung cancer patients and the serum prealbumin level can be used as a biomarker to predict early recurrence of lung cancer [27]. Besides, serum prealbumin level has also been confirmed as an independent prognostic factor for the patients of postoperative esophageal squamous cell carcinoma [28], liver cancer[29], and gastric cancer [30]. Notably, the practical markers cannot be found in time during the global outbreak of Coronavirus (COVID-19) infection in 2020. Many researchers have had to shift their focus to routine blood tests in the hope of finding cheap and accessible tests [31–33]. Brandon et al. found that some hematologic, biochemical, and immunologic biomarkers have discriminative ability. These clinical laboratory test items include interleukins 6 (IL-6) and 10 (IL-10) and serum ferritin which all potential aid in predicting severe and fatal COVID-19 were identified [34]. Moreover, other clinical studies also have shown significant changes in blood parameters in patients with COVID-19. These clinical laboratory items, include lactate dehydrogenase (LDH), white blood cell (WBC), C reactive protein (CRP), aspartate transaminase (AST), and alanine transaminase (ALT), which can play a crucial role in COVID-19 diagnosis and prognosis [35].

### Clinical significance of combined blood test items

Over the past several decades, the combinations of clinical laboratory test data also have gradually been applied to clinical diagnosis and treatment choices. Clinical evaluations of disease progression have even used combinations of test items as a score. For example, many studies found that the change of the ratio of AST/ALT is not only an item of hepatocyte injury but suitable for more diseases. Zhou et al. have shown that the high AST/ALT ratio can increase the pathogenetic risk of prostate cancer [36]. Besides, studies have reported a significant association between the pre-treatment AST/ALT ratio and survival in oropharyngeal squamous cell carcinoma patients [37], non-metastatic renal cancer patients [38], urothelial carcinoma [39], and metastatic renal cell carcinoma [40]. The combined neutrophil-to-lymphocyte ratio (NLR) also has been confirmed that is an adverse prognostic factor in many diseases at present, especially malignant tumors, including gastric cancer [41], and colorectal cancer [42], and non-small cell lung cancer [43, 44]. And the score of clinical evaluation of disease development composed of some routine test items is also widely used. The modified Glasgow prognostic score (mGPS) is an inflammation-based prognostic score that consists of CRP and albumin (ALB). The score is not only an independent prognostic factor in early esophageal cancer patients [45] but is considered an essential prognostic indicator in a study on prognostic factors in colon cancer [46]. The control nutritional status (CONUT) score consists of serum albumin, cholesterol, and total lymphocyte count. It is associated with the postoperative survival of patients undergoing hepatectomy [47] and gastric cancer resection [48], and can predict the survival of patients with hypertension over 80 years old [49]. It is not difficult to find that the combination of inexpensive, available and routine clinical test items seems to play an increasingly important role in clinical diagnosis and treatment.

## Combining AI with clinical laboratory

AI is a field of computer science that is designed to mimic human thinking processes, learning abilities, and knowledge storage [50]. In the age of big data, AI technology can use sizeable clinical data sets to support clinical decisions, uncover occult disease subtypes, associations, and prognostic indicators, and generate new testable hypotheses. AI is gradually changing the way that doctors make clinical decisions and diagnoses. AI has now been applied to several aspects of medicine, from diagnostic applications in radiology and pathology [51, 52] and the classification of various eye diseases in ophthalmology [53] to more therapeutic and interventional applications in cardiology and surgery [54, 55].

Machine learning (ML), is a significant branch of AI, and one of its advantages is learning from data [56, 57]. ML and deep learning (DL) techniques can handle large, complex, nonlinear, and multidimensional data better than conventional statistical methods [58, 59]. The development of clinical laboratory automation and the unification of data standardization have gradually transformed the clinical laboratory department into a large and credible clinical database in medicine. In addition, in the clinical laboratory department and the potential diagnostic value of clinical testing data and the value of the joint diagnosis of clinical testing items are gradually explored [60]. Therefore, the multifaceted development of clinical laboratories and the development of AI provide the conditions for their combination in the era of big data [61].

**Fig. 4:**
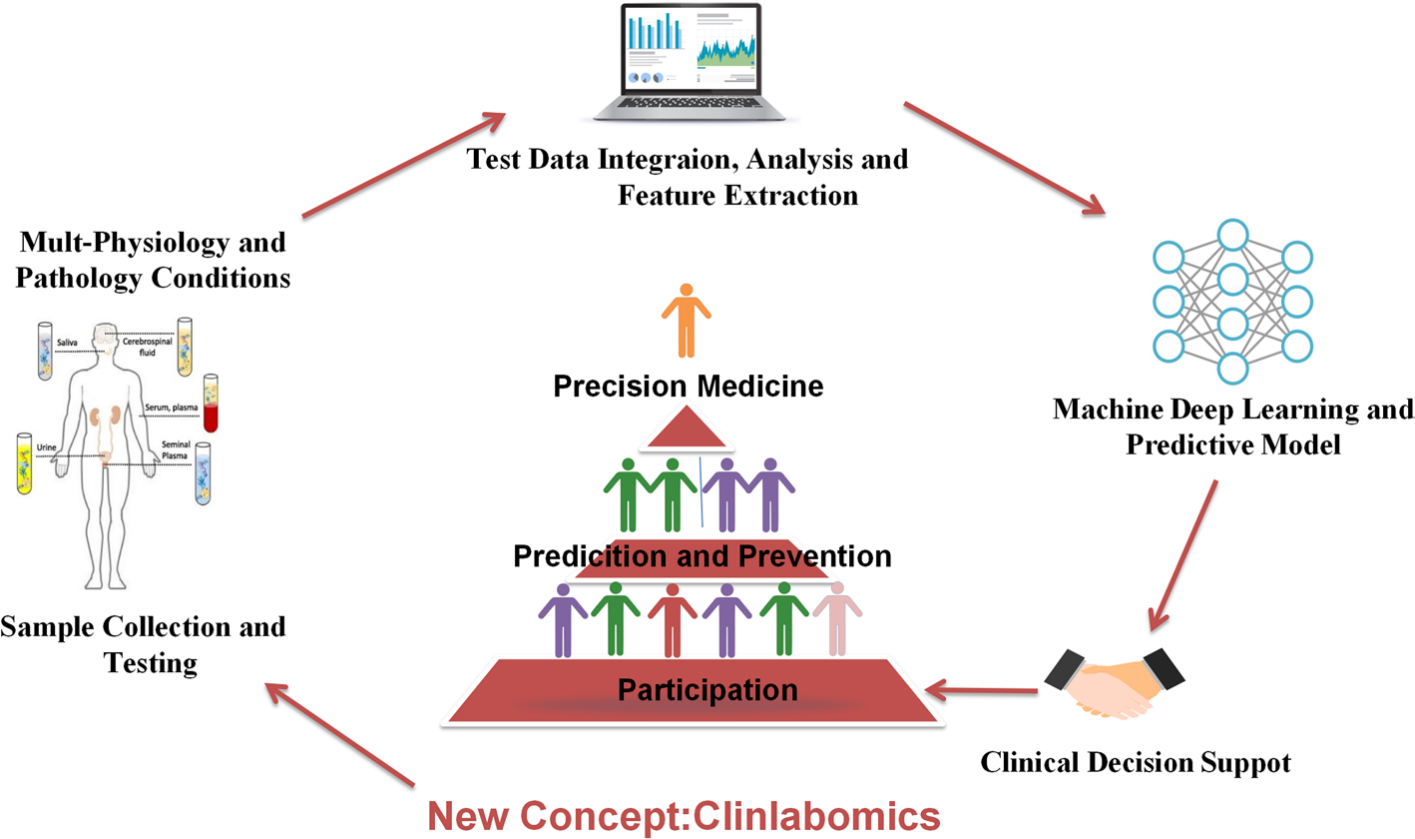
The Clinlabomics workflow. Collecting blood or body fluid sample and testing. From this clinical laboratory data to extract the features e.g. features based on range of clinical test data from healthy or patient with various diseases. These features are used for analysis, e.g. the features are assessed for their diagnostic prognostic power or linked with stage. Ultimately, it could lead to precision medicine and personalized medicine

From routine blood or body fluid test data, Clinlabomics extracts, analyzes, screens, and identifies certain reproducible and prominent clinical laboratory test indexes for patients with clinically relevant diseases. A relationship is then analyzed between the selected characteristic test items and the diagnosis and treatment results. Through in-depth ML of a large amount of data and the establishment of predictive models for related diseases, the aim is to provide accurate disease diagnosis, risk stratification, and prognosis (Fig. 4). Analysis of clinical laboratory test data can provide additional information not currently available. Furthermore, Clinlabomics can evaluate the added value of routine laboratory test items to common predictors of related diseases. The use of Clinlabomics can reduce unnecessary expenses and the time for diagnosing and treating clinical diseases. However, with a lack of such definitions as transcriptomics, metabolomics, radiomics, and other complete keywords, many research projects cannot be classified very well, which does not support the development of the field. We propose a concept of Clinlabomics to summarize some of these studies for diagnosing, treating, and predicting diseases by using clinical laboratory data along with AI.

At present, using clinical laboratory testing data combined with AI to perform disease diagnosis, prediction, monitoring, and prognosis research is booming [62, 63]. In the following sections, we summarized some relevant studies obtained using Clinlabomics (Table 1).

**Table 1:** The partial representative research of the application of Clinlabomics

Application fields	Year	Sample size	Best models of analysis	Objective and achievement
Clinical prediction	2019 [64]	149,000 physical samples	Deep Neural Network (DNN)	Biological aging prediction
	2016 [65]	62,419 physical samples	Deep Neural Network (DNN)	Biological aging and Smoking status prediction
	2021 [71]	285,965 diabetes patients and 1,221,598 healthy human samples	Extreme Gradient Boosting (XGBoost)	Risk prediction for diabetes
	2017 [72]	79 paraquat poisoning patients (41 living and 38 deceased)	Support Vector Machine (SVM)	Predicting the prognosis of paraquat poisoning patients
	2020 [73]	235 patients (89 benign ovarian tumors and 146 ovarian cancer)	Decision Tree Model	Predicting ovarian cancer
	2021 [80]	1823 COVID-19 patients	Extreme Gradient Boosting (XGBoost)	Predicting the mortality of patients with COVID-19
Clinical diagnosis	2012 [75]	203 iron deficiency anemia patients	Artificial Neural Network (ANN)	Iron deficiencyanemia diagnosis and iron serum level prediction
	2020 [76]	355 asthma patients and 1,480 Healthy individuals	Mahalanobis–Taguchi System (MTS)	Asthma diagnosis
	2019 [77]	551 chronic kidney disease patients	Logistic Regression Model (LR)	CKD severity diagnosis and surveillance
	2020 [79]	177 positive subjects and 102 negative subjects	Random Forest (RF)	COVID-19 infection diagnosis
	2019 [81]	15,176 Neurological patients	The Smart Blood Analytics (SBA) Machine Learning (ML) Algorithm	Brain tumors diagnosis
	2019 [82]	183 lung cancer patients and 94 patients without lung cancer	Random Forest (RF)	Lung cancer diagnosis
	2021 [83]	1168 colorectal cancer patients and 1269 healthy subjects	Logistic Regression Model (LR)	Colorectal cancer diagnosis
Clinical labortory management	2018 [85]	10,799 training samples and 9839 testing samples	Support Vector Machine (SVM)	Identifying wrong blood in tube errors prior to test reporting
	2021 [86]	141,396 samples	Artificial Neural Network (ANN)	Identifying mislabeled samples
	2021 [90]	192 clotted samples and 2889 normal blood samples	Back Propagation Neural Network (BPNN)	Identifying clotted specimens in coagulation testing
	2018 [91]	4619 samples of urine steroid profiles	Tree-based Model	Aiding the Interpretation of urine steroid profiles
	2022 [92]	202 consecutive chronic lymphocytic leukemia patients	Deep Neural Network (DNN)	Improving flow cytometry workflow efficiency for detecting of minimal residual disease of chronic lymphocytic leukemia
	2022 [95]	254 healthy samples, 8800 physical examination population and 7700 outpatient samples	Normally distributed data: Transformed Hoffmann, Transformed Bhattacahrya, Kosmic and RefineR Algorithms Data with obvious skewness: Expectation Maximization (EM) Algorithm combined with Box-Cox Transformation	Establishing reference intervals for thyroid-related hormones in older adults
	2019 [99]	212,554 urine samples	Extreme Gradient Boosting (XGBoost)	Screening urine microbiological inoculation samples

## The application of clinlabomics

### Clinlabomics and clinical prediction

The prediction of biological aging has been widely concerned. However, there are currently no informative tests to assess the impact of smoking on biological aging rates. Researchers collected data from 149,000 fully anonymized individual records. They trained a set of supervised feed-forward deep neural networks (DNNs) on the non-smokers to predict their chronological age. Then, they included smoking status as one of the input features and performed a feature importance analysis. Eventually they trained a set of supervised feed-forward deep neural networks to predict the smoking status of patients using only their sex and blood feature, including 66 kinds of blood biochemistry items and cell count markers. The model demonstrated that smoking accelerates human aging, and that smoking status could also be predicted from blood biochemical and cell count results. Although this blood aging clock model proved to be less accurate than predictors based on DNA methylation, it is cheaper and more practical, and only involves standard blood tests [64]. Additionally, Putin designed a modular ensemble of 21 DNNs of varying depth, structure and optimization to predict human chronological age using a basic blood test and used over 60,000 samples from common blood biochemistry and cell count tests from routine health exams to train DNNs. The best performing DNNs achieved an accuracy of 81.5% when testing human chronological age. Moreover, they found that albumin, glucose, alkaline phosphatase, urea, and red blood cells were five of the most important markers of predicting chronological age [65].

Clinlabomics also can predict many diseases, including cancer [66–69]. As everyone knows, diabetes is a global epidemic, chronic and incurable and long-term exposure to hyperglycemia can cause chronic damage to various tissues [70]. Early prediction can drastically reduce the risk of diabetes occurrence. Yang collected 1,507,563 physical examination data from healthy individuals and diabetes patients, as well as 387,076 physical examination data from the follow-up records. They fused three types of physical examination data: laboratory values (fasting blood glucose (FBG), high-density lipoprotein (HDL), low-density lipoprotein (LDL), serum creatinine (SC), triglyceride (TG), total cholesterol (TC), blood urea nitrogen (BUN), urine glucose (UGLU)) demographics, and vital signs in their computational model. They used mutual information, analysis of variance and Gini impurity to rank the features, and then, the incremental feature selection strategy was combined with XGBoost. Finally, they created a diabetes risk assessment model with high accuracy in detecting diabetes (AUC = 0.8763)[71]. This study result showed that the application of Clinlabomics could help the high-risk group take medicine or change lifestyle timely and reasonably so they can effectively reduce the risk of diabetes and prevent diabetes effectively. Besides, Chen developed a method based on a support vector machine combined with the blood routine indexes feature selection technique to accurately predict toxic paraquat (PQ) poisoning risk status. The results showed that there are significant differences in blood routine indexes between dead and living PQ-poisoned individuals (*p* value < 0.01) and the most important correlated indexes are WBCs and neutrophils [72]. Therefore, the toxicity or prognosis of PQ poisoning can be preliminarily predicted by blood routine testing. Finally, a simple decision tree model was constructed by applying the Minimum Redundancy-Maximum Relevance feature selection method to the 235 patients' data (89 benign ovarian tumors and 146 ovarian cancer samples). The results demonstrated that the decision tree model had strong predictive power for distinguishing ovarian cancer from benign ovarian tumors, and human epididymis protein 4 (HE4) and carcinoembryonic antigen (CEA) were valuable markers for ovarian cancer prediction [73]. Clinlabomics has good potential for providing predictive models for complex diseases, using the cheaper and more practical standard blood tests to support some clinical predictions.

### Clinlabomics and clinical diseases diagnosis

Clinlabomics not only plays a great role in clinical prediction, but also plays a significant role in clinical diagnosis. Recently, the research on the application of Clinlabomics in the diagnosis of clinical diseases has gradually increased. Muhsen summarized the application of ML in the field of hematology diagnosis, including Clinlabomics [74]. Azarkhish developed an artificial neural network (ANN) and an adaptive neuro-fuzzy inference system (ANFIS) to diagnose iron deficiency anemia (IDA) and to predict serum iron levels based on four accessible laboratory data (Mean corpuscular volume (MCV), Mean corpuscular hemoglobin (MCH), Mean corpuscular hemoglobin concentration (MCHC), Hemoglobin /red-blood-cell (Hb/RBC)) [75]. The ANN was the best model for diagnosing IDA with an accuracy of 97% for patients with IDA and 96% for patients without it.

Zhan used 14 routine blood test data (basophil count, eosinophil count, lymphocyte ratio, lymphocyte count, mean corpuscular hemoglobin, mean corpuscular hemoglobin concentration, monocyte ratio, monocyte count, mean platelet volume, platelet distribution width, platelet count, red blood cell count, red blood cell distribution width, and white blood cell count) from healthy individuals to construct a Mahalanobis space (MS). To ensure the efficiency of MS, they calculated Mahalanobis distances of blood data from 355 asthma patients and 1480 healthy individuals. Orthogonal arrays and signal-to-noise ratios were used to optimize blood biomarker variables the receiver operating characteristic (ROC) curve was used to determine the threshold value. Ultimately Mahalanobis-Taguchi system (MTS) correctly classified 94.15% of patients. In addition, 97.20% of healthy individuals were correctly classified [76]. Due to there being no gold standard for asthma diagnosis currently, we can see that the use of Clinlabomics offers the potential to simplify diagnostic complexity and optimize clinical efficiency.

Chronic kidney disease (CKD) severity can be assessed using urine protein concentration, but it can be inconvenient to collect 24-h urine for follow-up. 9 models were developed and compared using 13 routine blood test indexes and five demographic characteristics. These models as non-uretic clinical variables combine statistical, machine learning, and neural network methods to predict urinary protein progression in patients with chronic kidney disease. Their results showed that linear models including Elastic Net, lasso regression, ridge regression and logistic regression have overall predictive power, with an average AUC and precision above 0.87 and 0.8, respectively. Among them, LR obtained the highest AUC value of 0.873 [77].

Clinlabomics is also extremely important for detecting COVID-19 [78]. Based on hematochemical values from routine blood tests, Brinati developed two machine-learning classification models whose accuracy ranges between 82 and 86%, and sensitivity ranges between 92 and 95% [79]. Besides, Domínguez-Olmedo also developed a model to predict the mortality of patients with COVID-19, which can assess mortality from laboratory values with a high degree of accuracy [80].

In addition, Clinlabomics also plays a vital role in the diagnosis of some cancers. Simon used the routine blood tests from 15,176 neurological patients via the smart blood analytics (SBA) ML algorithm to build a machine learning predictive model for brain tumor diagnosis. Moreover, they validated the model by retrospective analysis of 68 consecutive brain tumors and 215 control patients presenting to the neurological emergency service. The sensitivity and specificity of the adapted tumor model in the validation group were 96% and 74%, respectively [81]. That result demonstrated the feasibility of brain tumor diagnosis by routine blood tests combined with machine learning. At the same time, it proved that the application of Clinlabomics can compensate for the low accuracy and expensive disadvantage of computed tomography (CT) imaging in the diagnosis of brain tumors. Similarly, Wu used a random forest machine-learning algorithm to build an identification model between routine blood indexes and lung cancer. A correlation between 19 regular blood indexes and lung cancer patients was found, and lung cancer patients could be identified from other patients, especially those with tuberculosis (which has similar symptoms to lung cancer), with a sensitivity of 96.3%, specificity of 94.97%, and accuracy of 95.7% for the cross-validation results, respectively [82]. Li also used laboratory data, including liver enzymes, lipid profiles, complete blood counts, and tumor biomarkers to develop five machine learning models to identify colorectal cancer (CRC). The results showed that the logistic regression model achieved the highest performance in identifying CRC (AUC: 0.865, sensitivity: 89.5%, specificity: 83.5%, PPV: 84.4%, NPV: 88.9%) [83]. Studies of Clinlabomics in diagnosing disease have increased, both in general and severe diseases, and have achieved remarkable diagnostic results. In addition to facilitating more convenient and accurate diagnostic methods, it also decreases the cost of diagnosing related diseases. In the age of big data, we can see that Clinlabomics is becoming more and more important for precision medicine. The routine blood test results contained much more information than is usually recognized even by the most experienced clinicians.

### Clinlabomics and clinical laboratory management

Clinlabomics also correctly conducts laboratory management to some extent, including laboratories formulate reference ranges, clinical laboratory quality control, and automated interpretation of laboratory testing results. Clinlabomics has the potential to improve laboratory efficiency and quality in a setting of limited staff resources [84]. One concerning the type of preanalytic error in laboratory medicine is the wrong blood in the tube (WBIT) error because blood specimens collected from one patient occasionally get mislabeled with identifiers from another patient. Continuous monitoring of specimen acceptability, collection and transport can result in the prompt identification and correction of problems, leading to improved patient care and a reduction in unnecessary redraws and delays in reporting results [85–88]. Rosenbaum simulated WBIT errors within sets of routine inpatient chemistry test results to develop, train, and evaluate five machines learning based WBIT detection algorithms*.* The results showed a best-performing WBIT detection algorithm based on a support vector machine to identify WBIT errors before test reporting. This algorithm achieved an area under the curve of 0.97 and considerably outperformed traditional single-analyte delta checks [85]. For evaluating the performance of identifying mislabeled samples, Farrell developed eight different machine learning models using different algorithms: artificial neural networks, extreme gradient boosting, support vector machines, random forests, logistic regression, k-nearest neighbors, and two decision trees (one complex and one simple). Moreover, it was compared with the ability to manually identification of mislabeled samples. The best performing machine learning model, the artificial neural network (92.1% accuracy), outdistanced human performance for identifying mislabeled samples(77.8% accuracy) [86]. Serum quality is also a key consideration in the pre-analytical phase of a laboratory analysis [89]. Fang retrospectively retrieved the coagulation test results (Activated partial thromboplastin time (APTT), Prothrombin time (PT), Thrombin time (TT), Fibrinogen (Fbg), and D-dimer) of 192 clot samples and 2889 clot-free test (NCD) samples to form a training and test dataset. Standard and momentum back-propagation neural networks (BPNNs) were trained and validated using training datasets and five-fold cross-validation methods to verify the feasibility of identifying clot specimens through machine learning. Surprisingly, the result confirmed that the standard and momentum BPNNs could identify the sample status (clotted and NCD) with areas under the ROC curves of 0.966 (95% CI 0.958–0.974) and 0.971 (95% CI 0.9641–0.9784), respectively [90].

Auto verification and auto-explanation systems might have greatly improved laboratory efficiency. Wilkes retrospectively collected 4619 urine steroid profile data to train and test various ML classifiers’ abilities to differentiate profiles. The results showed the best performing binary classifier could predict the interpretation of profiles with a mean area under the ROC curve of 0.955 (95% CI 0.949–0.961). In addition, the best performing multiclass classifier could predict the individual abnormal profile interpretation with a mean balanced accuracy of 0.873 (0.86–0.880) [91]. This provided a proof-of-concept application of ML algorithms to complex clinical laboratory data. Salama developed deep neural networks (DNN) to improve the efficiency of clinical laboratories in detecting minimal residual disease (MRD) in chronic lymphocytic leukemia (CLL) by flow cytometric immunophenotyping. The result showed that there was an excellent correlation between their DNN and expert analysis when CLL cells were reported as a percentage of total white blood cells. In addition, gating time was dramatically reduced to 12 s/case by DNN from 15 min/case by the manual process. The proposed DNN demonstrated high accuracy in CLL MRD detection and significantly improved workflow efficiency [92].

In addition, reference intervals are critical for the interpretation of laboratory results and Clinlabomics also can help to establish the reference interval [93, 94]. Ma validated five data mining algorithms using thyroid-related hormones test data from clinical laboratories to establish reference intervals of thyroid hormones for older adults. The results showed that the transformed Hoffmann, transformed Bhattacahrya, Kosmic, and refineR algorithms were the more suitable algorithms to establish reference intervals for thyroid-related hormones in older adults and an Expectation maximization (EM) algorithm combined with Box-Cox transformation was recommended for data with obvious skewness [95]. Poole developed LIMIT, an unsupervised learning method to extract reference intervals from the electronic medical record. Results showed that LIMIT produces usable reference intervals for sodium, potassium and hemoglobin laboratory results. From the above research, we conclude that Clinlabomics represents a fast and inexpensive solution for calculating reference intervals, and showed that it is possible to establish reference intervals by using laboratory results and AI [96].

The urine samples from patients suspected of urinary tract infection (UTI) generate the highest workload in routine clinical microbiology diagnostic laboratories [97]. However, the actual situation was that many urine samples produce negative culture results. There were no significant bacterial isolates or mixed culture results indicating sample contamination [98]. The reduction in the number of suspect samples that must be cultured will allow diagnostic services to focus on actual microbial infections, which will reduce the workload in the laboratory. Burton retrospectively analyzed a total of 212,554 urine microscopy, culture, and sensitivity urine reports. He compared the two classification methods: a heuristic model using a combination of white blood cell count and bacterial count and a machine learning approach testing three algorithms (Random Forest (RF), Neural Network (NN), and Extreme Gradient Boosting (XGboost)). The clinical laboratory items included in the machine learning approach include urine items of microscopic analysis, and biochemical dip-stick testing such as NIT, WBCUF, EC, and haematuria. Based on their initial findings, the machine learning algorithms outperformed the heuristic model in terms of relative workload reduction at a classification sensitivity above 95%. Using this method has a potential decrease of about 41% in the cultivation workload. XGboost achieved the highest AUC of 0.910among the three machine learning approaches [99].

From the above research, we concluded that Clinlabomics can help with clinical laboratory management and improve the efficiency of clinical diagnosis. Besides, Clinlabomics also may improve service efficiency when demand exceeds the resources of public health service providers.

## The challenge and opportunity of clinlabomics

The 2016 World Economic Forum listed the open AI ecosystem as one of the top 10 most important emerging technologies [100]. Since 2017, China, the United States, and the European Union have successively issued national-level artificial intelligence (AI) strategic development plans, in the field of clinical laboratory testing, the explosive growth of AI theories and technologies also provides a new direction for the development of medical testing theory, methods and applications [101–104].

By reviewing some recent studies of ML applications in the field of clinical laboratory medicine. It was not difficult to find that Clinlabomics in the clinical laboratory can conduct more rapid and efficient analytical processing of complex detection data. Not only that, Clinlabomics can correctly conduct laboratory management to some extent, which can play an important role in the future development and construction of laboratory medicine. Besides, Clinlabomics will certainly go further beyond its current boundaries in the field of clinical laboratory medicine. Just as during the global period of COVID-19 spread, Clinlabomics can further expand the scope of disease diagnostic tools, which is particularly promising to make up for the lack of skilled laboratory staff and adequate testing instruments in developing countries [32, 35, 79, 80, 105].

It is undeniable that the development of artificial intelligence has brought opportunities to the development of Clinlabomics but there are still a series of challenges and problems in the development process of Clinlabomics [106, 107].

On the one hand, although the current Clinlabomics to improve the efficiency of clinical laboratory testing and supplementary diagnosis of clinical diseases has great potential [108], a lot of clinical laboratory technicians for big data age and AI combined with clinical laboratory test data understanding is not deep [109]. In addition, the replacement of human labor with technological development has caused panic in the whole society, and clinical laboratory personnel also have the same concern [14, 109]. The clinical laboratory staff has not willing to further study and develop Clinlabomics resulting laboratory’s lack of bioinformatics professional knowledge, leading to this field is limited and the development slow. On the other hand, ML models rely on the type and quality of the data used for training, and often tend to perform better on data from the same cohort than on the new data. Different regions, different people, and even different hospitals' laboratory equipment, and methods may result in instability in Clinlabomics-related diagnosis models. The development of Clinlabomics requires the standardization of testing methods and data for each region, each country, each species, and each laboratory. Biological variation data and external validation is a necessary practice in Clinlabomics evaluation [110–113]. Besides, laboratory medicine, like other areas of medicine, is obliged to adhere to high ethical standards [114–117]. Informed consent is essential to maintaining patient autonomy [118]. However, it is sometimes difficult to balance patient autonomy with the idea of contributing to the development of medicine [119]. The use of remaining or stored samples is essential for research and the development of Clinlabomics, but it creates problems with consent. There is no doubt that this is a huge project for the current situation.

## Discussion and conclusion

In general, routine clinical laboratory test results usually contain more information than is usually recognized. Even the most expert clinicians are challenged to extract all the information contained in routine clinical laboratory tests [60, 63]. According to the relevant representative research reports in this review, it is not difficult to see that combining AI and clinical laboratory data has been applied, including disease prediction, diagnosis, and monitoring of disease status. Besides, it also is very conducive to laboratory management. Therefore, our proposed Clinlabomics is a new concept aimed at collecting valuable information obtained from routine laboratory tests. Although the research of combining AI and clinical laboratory data is still in its infancy, most studies focus on details information on routine testing items in the blood. From some research we summarized, combining blood-related testing items data and AI have achieved some results in the diagnosis, monitoring, and prognosis evaluation of clinical diseases and their conclusions. Many authors presented opportunities related to combining clinical laboratory data and AI methods, and some also made their algorithms available. However, extensive data clinical trials are still lacking to verify and the establishment of standardization. On the other hand, there are few studies combining AI with body fluid-related detection items (such as urine, and cerebrospinal fluid) in the clinical laboratory. In the future, we can carry out disease diagnosis and treatment-related research on body fluid-related detection items through the deep learning method. In addition, there are many studies combining AI and other medical fields, especially imaging and pathology. Therefore, using patient clinical information and laboratory data, combining data from other diagnostic facilities (such as pathology and radiology) and pharmacies) has the potential to further improve the accuracy and reliability of the diagnostic model.

As seen in the previous section, many studies use different models for comparison, the best model or algorithm used in combining AI and clinical laboratory data is different whether in disease prediction and diagnosis or laboratory management. Clinical laboratory data must be analyzed with appropriate models and algorithms to solve different problems. Logistic regression (LR) is one of the traditional models, its clarity, simplicity and great interpretability of the model are the reasons why LR was frequently chosen [63]. However, due to the simple form of the LR model (very similar to the linear model), it is difficult to fit the real distribution of data, so the accuracy is not high. Therefore, the LR model is currently widely used to predict the factors of disease pathogenesis [77, 83]. We can use the LR model to analyze and predict disease risk through clinical laboratory testing items. We also found that the Random Forest (RF) model was frequently used in studies we reviewed [73, 79, 82]. From a technical point of view, RF is an ensemble algorithm that relies on a collection of decision trees that are trained on mutually independent subsets of the original data to obtain a classifier with lower variance and/or lower bias [79]. This class of models also has generally high accuracy as well as interpretable output [63]. These are some reasons why RF is chosen. As a result of its merits, especially high accuracy, we think the RF model may be suitable for some applications related to disease diagnosis. A support vector machine (SVM) is a dichotomous model that is supported by strict mathematical theory and has strong explanatory power. It does not rely on statistical methods, thus simplifying the usual classification and regression problems. SVM has been applied to myriad classification tasks and has been demonstrated to be particularly effective for medical diagnosis [72]. Studies have reported that NN models (such as ANNs and DNNs) are widely used in control and optimization, prediction and management, pattern recognition and image processing [63, 65]. Since NN models extract features automatically, they require more training resources (time and data volume) than traditional ML models [64, 65, 86, 92]. For this reason, we think NN models may be more effective at integrating data from different laboratories to extract feature test items.

In this article, there are a few limitations. Due to the differences in keywords, some related articles may be overlooked since our search query only contains words commonly used in the area we intend to study. In addition, we conducted our search only using PubMed and focused on nearly 10 years of related research. Finally, we only compared the performance of commonly used models in the research we reviewed. We did not discuss some of the less commonly used models specifically.

In conclusion, we believe Clinlabomics, with its advantages of low cost, effectiveness, avoiding unnecessary treatment, and toxicity risk can provide a new way for personalized medicine in the future. The potential of Clinlabomics, which applies machine learning to laboratory data for diagnostic and prognostic purposes deserves more attention from clinicians-scientists who wish to take advantage of this new computer-based pathology and laboratory medical support. In the future, the establishment of relevant databases through standardized and standard clinical test data features in various medical institutions will provide us with high-quality medical help for accurate diagnosis and treatment, thus taking a concrete step towards the realization of precision medicine.

## Data Availability

Not applicable.
